# Characterization of 3-phosphoglycerate kinase from *Corynebacterium glutamicum* and its impact on amino acid production

**DOI:** 10.1186/1471-2180-14-54

**Published:** 2014-03-04

**Authors:** Gajendar Komati Reddy, Volker F Wendisch

**Affiliations:** 1 Genetics of Prokaryotes, Faculty of Biology & CeBiTec, University of Bielefeld, Bielefeld 33615, Germany

**Keywords:** *Corynebacterium*, Homo dimeric Phosphoglycerate kinase, Glycolysis, Arginine production, Ornithine production, Amino acid productivity

## Abstract

**Background:**

*Corynebacterium glutamicum* cg1790/*pgk* encodes an enzyme active as a 3-phosphoglycerate kinase (PGK) (EC 2.7.2.3) catalyzing phosphoryl transfer from 1,3-biphosphoglycerate (bPG) to ADP to yield 3-phosphoglycerate (3-PG) and ATP in substrate chain phosphorylation.

**Results:**

*C. glutamicum* 3-phosphoglycerate kinase was purified to homogeneity from the soluble fraction of recombinant *E. coli*. PGK^His^ was found to be active as a homodimer with molecular weight of 104 kDa. The enzyme preferred conditions of pH 7.0 to 7.4 and required Mg^2+^ for its activity. PGK^His^ is thermo labile and it has shown maximal activity at 50–65°C. The maximal activity of PGK^His^ was estimated to be 220 and 150 U mg^-1^ with K_M_ values of 0.26 and 0.11 mM for 3-phosphoglycerate and ATP, respectively. A 3-phosphoglycerate kinase negative *C. glutamicum* strain *∆pgk* was constructed and shown to lack the ability to grow under glycolytic or gluconeogenic conditions unless PGK was expressed from a plasmid to restore growth. When *pgk* was overexpressed in L-arginine and L-ornithine production strains the production increased by 8% and by 17.5%, respectively.

**Conclusion:**

Unlike many bacterial PGKs, *C. glutamicum* PGK is active as a homodimer. PGK is essential for growth of *C. glutamicum* with carbon sources requiring glycolysis and gluconeogenesis. Competitive inhibition by ADP reveals the critical role of PGK in gluconeogenesis by energy charge. *Pgk* overexpression improved the productivity in L-arginine and L-ornithine production strains.

## Background

Central carbon metabolism uses a complex series of enzymatic steps to convert sugars into metabolic precursors [1]. When terminal electron acceptors are not available, glycolysis supplies all of the ATP molecules required for cellular activity through substrate chain phosphorylation and the glycolytic intermediates are direct precursors of many cellular building blocks. In glycolysis, substrate chain phosphorylation has a net yield of two moles of ATP per mole of glucose that are generated in the reactions catalyzed by 3-phosphoglycerate kinase (PGK) and pyruvate kinase. PGK catalyzes the phosphoryl transfer from 1,3-biphosphoglycerate (bPG) to ADP yielding 3-phosphoglycerate (3-PG) and ATP [2].

*C. glutamicum* is a Gram-positive soil bacterium belonging to the order *Corynebateriales* within the class of *Actinobacteria*[3]. Since the discovery of this organism, it has been used for the industrial production of L-amino acids, and strains have been developed for the production of D-amino acids, organic acids, diamines or biofuels from different carbon sources [4-8]. *C. glutamicum* was metabolically engineered for the production of amino acids from alternative substrates, such as starch, the hemicellulose components xylose and arabinose, cellobiose, the whey components galactose and lactose, and glycerol [9-11]. The phosphotransferase system substrates such as glucose, fructose and sucrose are used for production of amino acids on an industrial scale in the form of starch hydrolysate or molasses [12].

*C. glutamicum* has been extensively studied and the central carbon metabolism genes in *C. glutamicum* are under the control of a transcriptional regulatory network composed of several global regulators and various transcriptional regulators which have been characterized [13,14]. The genes of glycolytic enzymes are not clustered except for the *gapA-pgk-tpi-ppc* cluster encoding glyceraldehyde-3-phosphate dehydrogenase, phosphoglycerate kinase, triose phosphate isomerase and phosphoenolpyruvate carboxykinase, respectively [14]. The transcription of the *gapA-pgk-tpi-ppc* cluster involves mono-, di- and tricistronic mRNAs [15]. Expression of the *gapA-pgk-tpi* operon is coordinately regulated by SugR, RamA, and GlxR [16-18].

However, despite of the importance as an energy generating metabolic reaction in glycolysis in *C. glutamicum* and other bacteria, limited information is available on PGK. In this work, we aimed to identify and enzymatically characterize the PGK from *C. glutamicum* and to analyze its physiological role.

## Results

### Phylogenetic analysis of PGK from *C. glutamicum*

Databank searches with the amino acid sequences for PGK of *C. glutamicum* revealed similarities to biochemically characterized PGKs of different multimerization states such as monomeric PGK from *E. coli,* dimeric PGKs from *Methanothermus fervidus*[19] and *Pyrococcus woesi,* and tetrameric PGKs from *Sulfolobus solfataricus* and *Trypanosoma brucei*, the latter shown to be a dimer of dimers [20-23]. The N-terminal sequences of all PGKs showed high amino acid sequence similarity and a proline residue mostly conserved in all proteins in the hinge region. Based on its amino acid sequence, PGK from *C. glutamicum* is closely related to the monomeric enzyme from *E. coli* (Data not shown).

### Purification of PGK of *C. glutamicum* from recombinant *E. coli* expressing cg1790/*pgk*

**Figure 1:**
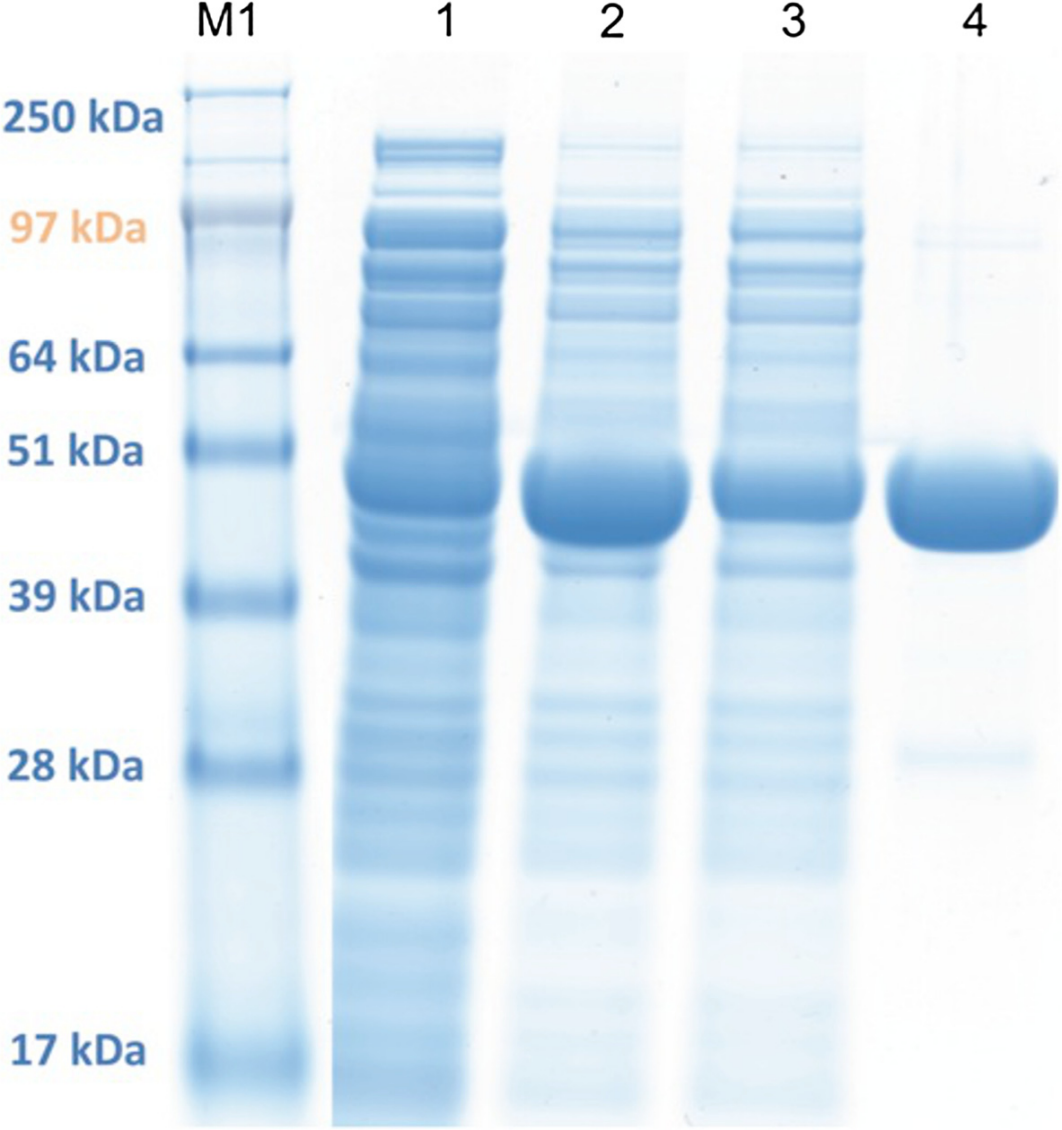
**Sodium dodecyl sulfate-polyacrylamide gel electrophoreses of expression and purification of PGK**^**His **^**of *C. glutamicum *from *E. coli *BL21 (DE3; pET16b-*pgk*).** The gel was loaded with cell extract from *E. coli* (pET16b-*pgk*) before (lane 1) and 4 h after induction with 0.5 mM IPTG (lane 2), the flow through after Ni-NTA chromatography (lane 3), and the eluate of Ni-NTA chromatography after rebuffering on a PD-10 column (lane 4). Lanes M1 shows protein standards SeaBlue Plus2 prestained standard (Invitrogen) containing protein of the indicated masses.

For purification, PGK from *C. glutamicum* was overproduced as an N-terminally His-tagged protein in recombinant *E. coli*. Metal chelate chromatography allowed to purify the enzyme to homogeneity as determined by SDS-PAGE (Figure 1) revealing a molecular mass of 47 kDa for the monomer. To determine the multimeric state of *C. glutamicum* PGK^His^, gel filtration chromatography was performed revealing a single peak with a molecular mass of about 104 kDa and only this fraction showed activity as PGK (data not shown). Thus, unlike the monomeric PGK from *E. coli,* PGK from *C. glutamicum* appears to be active as a homodimer as also reported for several archaeal PGKs [22].

### Characterization of PGK from *C. glutamicum*

The pH and metal ion concentrations were varied to determine the optimal buffer conditions for activity of PGK^His^. Within a pH range of 4.5 to 9.8, PGK activity was optimal at pH 7.0-7.4 in 100 mM TEA-Cl buffer with only 10% of PGK activity remaining at pH 4.5 and 33% at pH 8.9. PGK required bivalent cations with Mg^2+^ being the most effective. In the presence of 1 mM Ni^2+^, Co^2+^, Mn^2+^, Cd^2+^, Ca^2+^ or Zn^2+^, PGK^His^ activity was reduced to between 80% and 40% in comparison to the presence of Mg^2+^ only. To determine its thermal stability, PGK^His^ was incubated at temperatures ranging from 25 to 65°C prior to activity measurements at 30°C. A temperature optimum between 50-65°C was observed. Protein precipitation was observed above 65°C due to the instability of rabbit muscle GAPDH used as indicator enzyme in the assay.

### Kinetic parameters of PGK from *C. glutamicum*

The kinetic parameters of PGK^His^ for the substrates 3-phosphoglycerate and ATP were determined at 30°C, the optimal growth temperature of the bacterium. The activity of PGK^His^ with 3-phosphoglycerate and ATP in the gluconeogenetic direction followed Michaelis-Menten kinetics (data not shown). The K_M_ values of *C. glutamicum* PGK for 3-phosphoglycerate and ATP, respectively, were determined to be 0.26 mM and 0.11 mM, respectively. The Vmax values were 220 U mg^-1^ and 150 U mg^-1^, respectively (Table 1). Catalytic efficiencies were about 733 s^-1^ mM^-1^ and 592 s^-1^ mM^-1^, respectively (Table 1).

**Table 1:** Biochemical properties of PGK

**Parameter**		**PGK**
Molecular weight		47 kDa
		104 kDa (dimer)
Assay conditions		100 mM TEA-Cl, pH 7.4, 2 mM Mg^2+^, 0.2 mM NADH, 10 U/ml of GAPDH (rabbit muscle), 30°C
Optimal pH		7.0 - 7.4
Optimal temperature		55 - 60°C
Temperature stability		≥60°C
**Kinetics**		
ATP	K_M_	0.11 mM
	v_max_	150 U/mg
	k_cat_	130 s^-1^
	k_cat_/K_M_	592 s^-1^ mM^-1^
3-PG	K_M_	0.26 mM
	v_max_	220 U/mg
	k_cat_	191 s^-1^
	k_cat_/K_M_	733 s^-1^ mM^-1^

Values for K_M_ (mM), V_max_ (U/mg), and catalytic efficiency (K_cat_/K_M_ = s^-1^ mM^-1^) were determined for two independent protein purifications.

**Figure 2:**
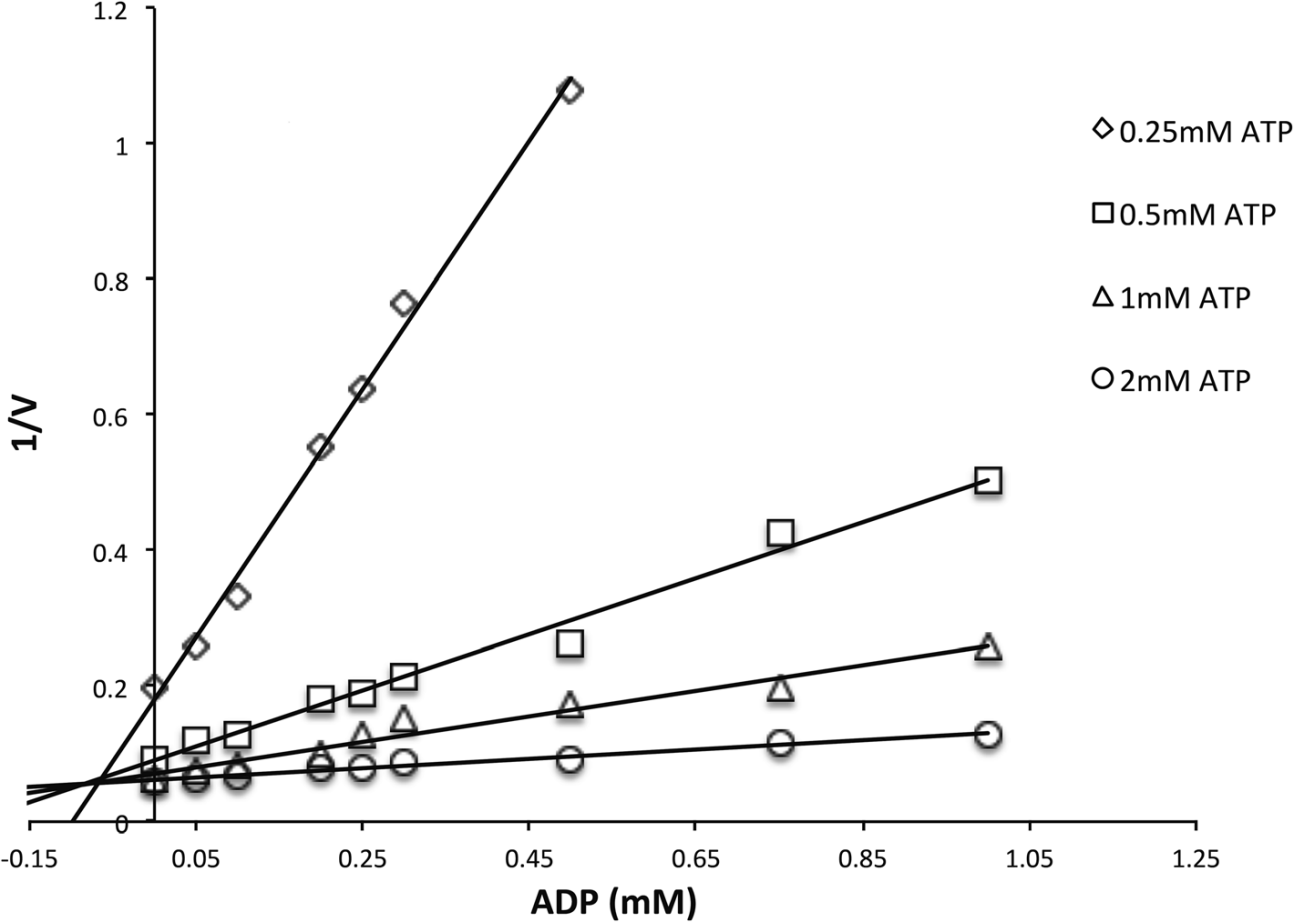
**Effect of ADP on the activity of 3-phosphoglycerate kinase at variable ATP concentrations.** The ATP concentrations were 0.25 mM (open diamonds), 0.5 mM (open squares), 1 mM (open triangles), 2 mM (open circles). The concentration of 3-PG was 10 mM through out the experiment. 1/v corresponds to reciprocal of the velocity of the reaction.

Metabolites such as glycerol-2-phosphate, glycerol-3-phosphate, glucose-6-phosphate, fructose-6-phosphate, fructose-1, 6-bisphosphate, phosphoenolpyruvate, pyruvate, acetate, L-lysine, L-alanine, L-glutamate, GTP, AMP and ADP were tested as potential effectors of PGK activity in the gluconeogenic direction (data not shown). Only ADP affected PGK activity as competitive inhibitor with 0.1 mM resulting in half-maximal activity (Figure 2).

### Physiological studies of *pgk* deletion mutants

*pgk* was deleted to study the role of PGK in *C. glutamicum*. Since *pgk* is part of the *gapA-pgk-tpi-ppc* operon [15] the specific activities of GapA, Tpi and Ppc were determined in crude extracts of the deletion mutant. While GapA activity in ∆*pgk* was comparable to wild type, ∆*pgk* showed decreased Tpi activity (0.1 U/mg as compared to 0.4 U/mg in wild type) and Ppc activity (0.03 U/mg as compared to 0.05 U/mg in wild type) which might be explained by polar effects of *pgk* deletion on the downstream genes *tpi* and *ppc*. Thus, plasmid pEKEx3-*pgk* was constructed for complementation analysis. *C. glutamicum ∆pgk* showed no growth in CgXII minimal medium with glucose or pyruvate as sole source of carbon and energy, but grew with blends of glucose plus pyruvate (Table 2). Complementation of *C. glutamicum ∆pgk* led to comparable growth with either glucose or pyruvate as sole carbon source (Table 2) revealing the requirement of PGK for growth with glycolytic as well as with gluconeogenic carbon sources.

**Table 2:** **Growth rates of *C. glutamicum *** **strains WT(pEKEx3), ∆ *pgk *** **(pEKEx3), *Cg∆pgk*** **(pEKEx3- *pgk *)**

**Carbon source**	***C. glutamicum***	***Cg∆pgk***	***Cg∆pgk*** **(pEKEx3- *pgk *)**
Glucose (100 mM)	0.32 ± 0.01	n.g.	0.36 ± 0.02
Pyruvate (200 mM)	0.30 ± 0.01	n.g.	0.28 ± 0.00
Glucose (5 mM) + pyruvate (50 mM)	0.24 ± 0.01	0.11 ± 0.01	0.23 ± 0.00
Glucose (5 mM) + pyruvate (100 mM)	0.31 ± 0.01	0.15 ± 0.01	0.32 ± 0.00
Glucose (5 mM) + pyruvate (200 mM)	0.36 ± 0.01	0.15 ± 0.01	0.33 ± 0.02

Data represent mean values and standard deviations of three independent replicates. n.g, no significant growth occurred.

### Effect of *pgk* overexpression on amino acid production

**Figure 3:**
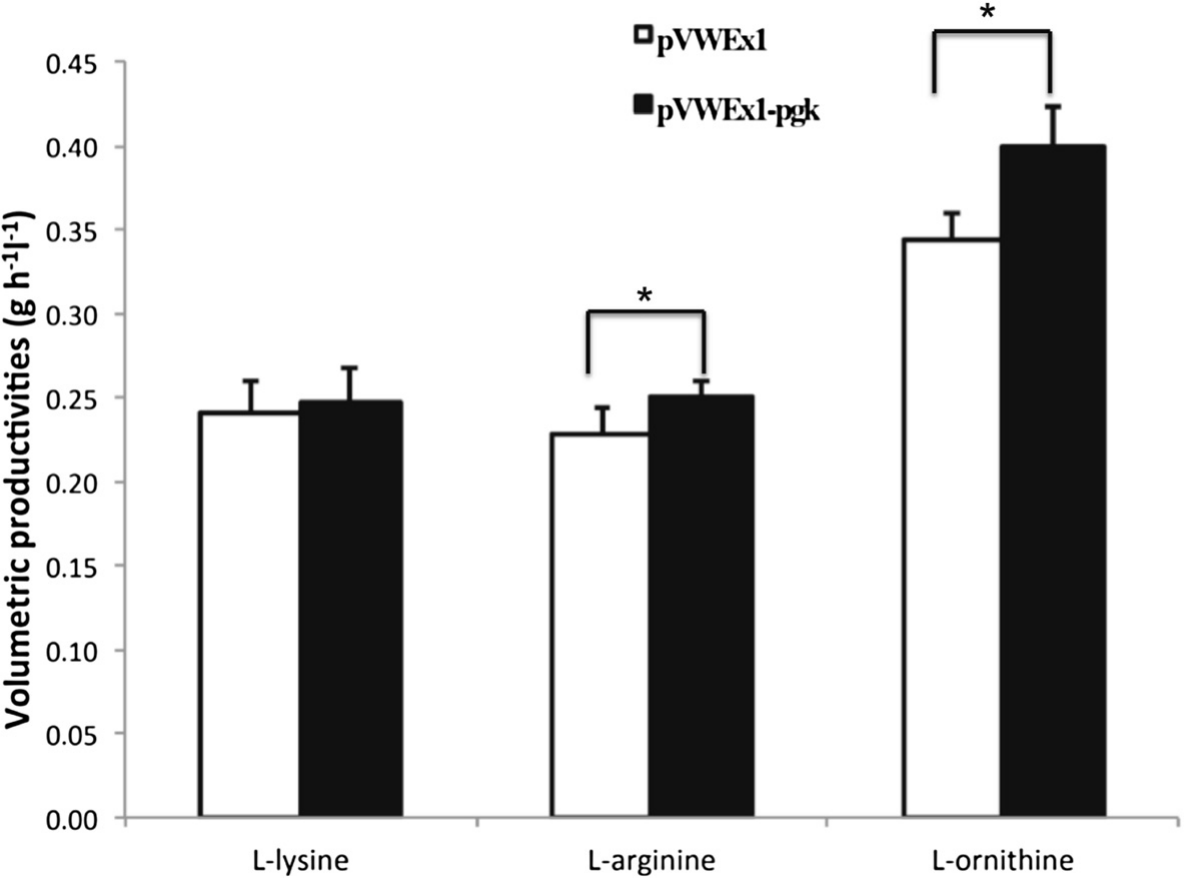
**L-lysine, L-arginine and L-ornithine production by *C. glutamicum *DM1933, ARG1 and ORN1 strains either with empty vector (white column represents pVWEx1) or overproducing phosphoglycerate kinase (black column represents pVWEx1-*pgk*).** Cells were grown in CgXII minimal medium with 4% glucose as a carbon source. Cell pellets of cultures grown in glucose CgXII minimal medium after consumption of the carbon source. Data represent mean values and standard deviations of six replicates from two independent cultivations with three flasks per strain each. Significant differences (p < 0.01 in a student’s *t*-test) between empty vector control (pVWEx1) and strains carrying pVWEx1-*pgk* are highlighted by an asterisk.

*pgk* was overexpressed in the L-lysine producing strain DM1933 [7], the L-arginine production strain ARG1 [6], and the L-ornithine production strain ORN1 [6] to assay the affects of *pgk* overexpression on amino acid production rates. In each strain, PGK specific activities increased upon *pgk* overexpression to similar levels as when *pgk* was overexpressed in the wild type (Table 3). Amino acid production in CgXII minimal medium with 4% (w/v) glucose was performed and amino acid accumulation was followed. While *pgk* overexpression did not increase the L-lysine production rate, *pgk* overexpression accelerated L-arginine and L-ornithine production by 8% and 17.5%, respectively (Figure 3). Glucose consumption was comparable between control and *pgk* overexpression strains.

**Table 3:** Phosphoglycerate kinase specific activity (U/mg)

**Strain**	**Specific activity (μmol/min/mg protein)**
WT(pEKEx3)	0.9 ± 0.08
WT(pEKEx3-*pgk*)	2.9 ± 0.2
WT(pVWEx1-*pgk*)	11.4 ± 0.9

Crude extracts were obtained by sonication of cells cultured in LB medium supplemnted with 1 mM IPTG and 25 μg/mL kanamycin.

## Discussion

Phosphoglycerate kinase from *C. glutamicum* was purified to homogeneity and shown to be active as homodimer. This is unusual and was hitherto only found in the thermophilic archaea *Pyrococcus* and *Methanothermus*[19,22]. Most prokaryotes and eukaryotes possess monomeric PGKs [24,25]. Tetramers have been reported in rare cases as in *Sulfolobus solfataricus*[26] and *Trypanosoma*[21]. *C. glutamicum* PGK^His^ showed higher temperature stability than monomeric PGKs, e.g., 40°C for *Mus musculus*[27] and 24°C for *S. cervisiae*[28]. Possibly, dimerization of PGK from *C. glutamicum* contributes to the stability of the protein by favoring hydrophobic interactions via the subunit contacts and by reducing the surface area exposed to the solvent in a similar manner as reported for dimeric PGKs [29]. Other characteristics are shared by many PGKs. An optimum pH 7.0 to 7.4 is found for phosphoglycerate kinases from *Escherichia coli* and *Homo sapiens,* while *Saccharomyces cerivisiae* shows optimum activity at pH 7.5 [24,30,31]. The K_M_ for ATP of PGK^His^ of 0.11 mM is similar to those reported for PGK from *Mus musculus* and *Homo sapiens*[32,33] and within the range reported for prokaryotic PGKs, e.g. 0.21 mM for PGK from *Pseudomonas sp*. and 0.31 mM for yeast PGK [33,34]. The K_M_ value of 0.26 mM for 3-PG of PGK^His^ is comparable to that of PGK from *Mus musculus*[33] and slightly lower than that of PGK from *Pseudomonas sp*. which is 0.48 mM [35]. The enzyme is very specific for Mg^+2^ ions for catalysis as reported for several other phosphoglycerate kinases. Other divalent cations such as Ni^2+^, Co^2+^, Mn^2+^, Cd^2+^, Ca^2+^ or Zn^2+^ could not replace Mg^2+^ and were inhibitory at higher concentrations. Zn^2+^ is a strong inhibitor with K_i_ of 0.45 mM. The monovalent cations Na^+^ and K^+^ activated the enzyme whereas sulfate ions showed no influence as reported for multimeric PGKs from archaea [22].

PGK from *C. glutamicum* was shown to be subject to allosteric regulation. Of the cellular metabolites tested only ADP affected the activity of PGK^His^. ADP inhibited PGK from *C. glutamicum* as competitive inhibitor with a low K_i_ value (0.1 mM). Since ADP concentrations in *C. glutamicum* typically range from 0.5 to 1 mM in glucose batch cultures [36], this indicates that gluconeogenesis via PGK is tightly regulated by the energy charge. Regulation by the energy charge has also been described for eukaryotic PGKs, such as mouse PGK which has a very low K_i_ of ≈ 0.08 mM [32] and yeast PGK which has a K_i_ of ≈ 0.22 mM [37].

PGK from *C. glutamicum* was shown here to be required for growth with different glycolytic as well as with different gluconeogenic carbon sources. Besides PGK, other enzymes of glycolysis are expected to be essential for both glycolysis and gluconeogenesis in *C. glutamicum*, however, experimental evidence has only been obtained for fructose-1,6-bisphosphate aldolase [38] and phosphoglycerate mutase [39]. Deletion of the genes encoding phosphofructokinase and glyceraldehyde-3-phosphate dehydrogenase (GapA) [40] prevented growth with glycolytic substrates, while growth with gluconeogenic substrates was still possible. On the other hand, growth of *C. glutamicum* on gluconeogenic carbon sources has been shown to be dependent on PEPCk [41] and FBPase [42], but the lack of these enzymes did not preclude growth with glycolytic substrates. Deletion of the phosphoglucoisomerase gene *pgi* perturbed growth on glucose indirectly since the glucose PTS permease gene *ptsG* was hardly expressed in the deletion mutant. The lack of pyruvate kinase cannot be compensated during growth with some glycolytic or gluconeogenic carbon sources [43]. Pyruvate kinase is essential for growth with glycolytic non-PTS carbon substrates such as maltose but not for PTS substrates such as glucose, fructose and sucrose. Similarly, pyruvate kinase is essential for growth on gluconeogenic carbon sources that do not enter the central carbon metabolism via pyruvate, such as acetate or citrate, but is not required for growth with pyruvate or lactate [43].

Overproduction of PGK did not notably accelerate growth of *C. glutamicum* with glucose (data not shown). However, ornithine and arginine production rates were increased upon *pgk* overexpression in the respective amino acid producing strains (Figure 3), while lysine production was not accelerated. It is tempting to speculate that arginine and ornithine biosynthesis are positively affected while lysine biosynthesis is not because the latter has a lower ATP requirement (1 ATP per lysine) than the former (2 ATP per ornithine or arginine which in addition requires carbamoylphosphate). Most metabolic engineering approaches of glycolysis in *C. glutamicum* focused on increasing the product yield e.g. by redirecting carbon flux to reduce glycolysis and increase pentose phosphate pathway flux and NADPH provision [44-46]. Overexpression of the gene encoding gluconeogenic enzyme FBPase during growth and production with sucrose increased gluconeogenic flux from fructose-1, 6-bisphosphate to glucose-6-phosphate and into the pentose phosphate pathway [47]. Production of D-lactic acid [48], succinic acid [49] and alanine [50] by *C. glutamicum* under oxygen-deprivation conditions is characterized by a high glycolytic flux which could be enhanced by overexpression of *gapA.* Also overexpression of the genes coding for phosphofructokinase, triosephosphate isomerase, and fructose-1,6-bisphosphate aldolase accelerated D-lactate production under oxygen-deprivation conditions [48].

## Conclusions

*C. glutamicum* 3-phosphoglycerate kinase encoded by *pgk* was shown to be essential for growth with glycolytic as well as with gluconeogenetic carbon sources. ADP was shown to be a competitive inhibitor of PGK, which unlike most bacterial PGKs is active as a dimer. Since overexpression of *pgk* increased amino acid productivity for ornithine and arginine, but not for lysine, PGK may be a promising target to accelerate production processes requiring high glycolytic flux but needs careful testing.

## Methods

### Microorganisms and cultivation conditions

Microorganisms are listed in Table 4. ATCC 13032 was used as *C. glutamicum* wild type (WT) [51] along with the amino acid producing strains DM1933, ORN1, ARG1 [6], PUT21 [52]. *C. glutamicum* strains were pre-cultured in lysogeny broth (LB) medium [53] with antibiotics added when appropriate. *E. coli* strains DH5α [54] and BL21 (DE3) [55] were used as host for cloning and heterologous expression, respectively.

**Table 4:** List of bacterial strains and plasmids

**Strain, plasmid**	**Function and relevant characteristics**	**References**
***E. coli***		
DH5α	General cloning host (F^-^*thi-1 endA1 hsdR17*(*r*^*-*^ m^-^) *supE44* ∆lacU169 (^-^80lacZ∆M15) *recA1 gyrA96 relA1*)	BRL
BL21 (DE3)	Host for recombinant protein production (*ompT hsdSB*(*rB*^*-*^ mB^_^) *gal dcm* (DE3))	Novagen
***C. glutamicum***		
ATCC13032	WT strain, auxotrophic for biotin	[51]
Δ*pgk*	In-frame deletion of the *pgk* gene of WT	This work
DM1933	*Δpck pyc*(P458S) *hom*(V59A), 2 x of *lysC*(T311I), 2x of *asd*, 2x of *dapA*, 2 x of *dapB*, 2 x of *ddh*, 2 x of *lysA*, 2 x of *lysE*	[7]
ORN1	L-ornithine overproducing strain derived from WT, auxotrophic for L-arginine due to *argF* deletion	[6]
ARG1	*ΔargR* (pEKEx3-*argB*_*(*A26VM31V)_); in-frame deletion of *argR*	[6]
**Plasmids**		
pGEM-T	General cloning vector	Promega
pEKEx3	Spec^R^; *C. glutamicum*/*E. coli* shuttle vector (*P*_*tac*_, *lacI*^q^; pBL1, *OriV*_*C.g.*_, *OriV*_*E.c.*_)	[57]
pEKEx3-*pgk* (*Cg*)	Derived from pEKEx3, for regulated expression of *pgk* of *C. glutamicum*	This work
pVWEx1	Kan^R^, Ptac, lacI^q^	[58]
pVWEx1-*pgk*	Derived from pVWEx1, for regulated expression of *pgk* of *C. glutamicum*	This work
pET16b	Amp^R^; T7*lac*; vector for his-tagged protein overproduction	Novagen
pET16b-*pgk* (*Cg*)	Purification of his-tagged (His_6_) *C. glutamicum* PGK from *E. coli* BL21(DE3)	This work
pK19*mobsacB*	Km^R^; *E. coli*/*C. glutamicum* shuttle vector for construction of insertion and deletion mutants in *C. glutamicum* (pK18 *oriV*_*Ec*_*sacB lacZ*α)	[59]
pK19*mobsacB*∆*pgk*	pK19*mobsacB* with a *pgk* deletion construct	This work

For growth and amino acid production experiments, exponentially growing cells of LB precultures (50 ml) were harvested by centrifugation (3200 × g, 10 min), and washed in CgXII medium [56] without carbon source. Cultures of 50 ml CgXII media containing 4% (w/v) glucose, 100 μg/ml spectinomycin or 25 μg/ml kanamycin, and 1 mM IPTG were inoculated to a final optical density (OD_600_) of 1 and incubated in 500 ml baffled shake flasks at 30°C. The OD_600_ was measured in dilutions resulting in an OD_600_ between 0.05 and 0.25 using a Shimadzu UV-1202 spectrophotometer (Duisburg, Germany). For enzymatic activity determination in cell-free extracts, cells were grown in LB medium to mid-exponential phase (OD_600_ of 3.5 to 4), harvested by centrifugation (10 min at 3200 × g, 4°C) and washed in 100 mM TEA-Cl pH 7.4. Cells were stored at -20°C until usage.

### Overexpression of *pgk* in *C. glutamicum*

For overexpression of *pgk*, the gene was amplified via PCR from genomic DNA of *C. glutamicum* WT. The PCR was performed using the oligonucleotide primers listed in Table 5. To allow IPTG inducible expression of *pgk* in *C. glutamicum* the PCR-product was ligated into SmaI restricted vector pEKEx3 resulting in pEKEx3-*pgk* and XbaI restricted vector pVWEx1 resulting pVWEx1-*pgk*. Sequencing confirmed the integrity of the construct.

**Table 5:** Sequences of oligonucleotide primers

**Name**	**Sequence (5′-3′)**	**Function and relevant characteristics**	
*pgk*-Cgl-fw	*GATCTAGAGAAAGGAG*GCCCTTCAGATGGCTGTTAAGACCCTCAAGG	OE of *Cgl pgk*; start; RBS	
*pgk*-Cgl-fw	GATCTAGATTACTGAGCGAGAATTGCAACG	OE of Cgl *pgk*; stop;	
*pgk-*pur-fw	**CATATG**GATGGCTGTTAAGACCCTCAAGG	Purification of Cgl PGK, start; NdeI	
*pgk-*pur-rv	CATATGGATCTAGATTACTGAGCGAGAATT**GCAACG**	Purification of Cgl PGK, stop; NdeI	
*pgk*-Del-A	GACCTTCAACACCAAGTCTGAG	Del of *pgk*	
*pgk*-Del-B	*CCCATCCACTAAACTTAAACA*TGAGGGTCTTAACAGCCATGC	Del of *pgk*	
*pgk*-Del-C	*TGTTTAAGTTTAGTGGATGGG*CCCAGGCGTTGCAATTCTC	Del of *pgk*	
*pgk*-Del-D	CTTCGCAGCAACCAACTCATC	Del of *pgk*	
*pgk*_Del_ver_fw:	CATACACTGGCGACCAGC	Verification of *pgk* deletion	
*pgk*_Del_ver_rv	CTGCCTTAACAGAACCACCG	Verification of *pgk* deletion	

Restriction sites are highlighted in bold, linker sequences for crossover PCR and ribosomal binding sites are shown in italics. *Abbreviations:OE* overexpression, *Del* deletion, *RBS* ribosomal binding site, *CglC. glutamicum*.

### Overproduction of PGK in *E. coli*, protein purification and molecular weight determination

For heterologous expression of the 3-phosphoglycerate-kinase gene *pgk* (Cg1790) in *E. coli* BL21 (DE3), *pgk* was amplified via PCR from genomic DNA of *C. glutamicum* WT using the following oligonucleotide primers listed in Table 5. The 1208 bp amplification product was cloned into vector pGEM-T (Promega, Mannheim, Germany) resulting in vector pGEM-T-*pgk*. After restriction with NdeI, the 1208 bp product from pGEM-T-*pgk* was ligated to NdeI restricted pET16b (Novagen, Madison, WI, USA). The vector, pET16b-*pgk*, allows IPTG-inducible expression of an N-terminal tenfold His-tagged *pgk* in *E. coli* BL21 (DE3). Cultivation of BL21 (DE3) (pET16b-*pgk*), Cells were grown in an incubator/shaker at 37°C to an absorbance reading (A_600_) of 0.6–0.8 at which point IPTG (0.5 mM) was added and the flasks were cooled to 22°C and further incubated for 4 hours. Cells were harvested by centrifugation (20 min at 3200 × g) and the cell pellet was washed with 20 mM Tris, 300 mM NaCl, 5 mM imidazole (TNI buffer), 5% (vol/vol) glycerol and stored at -80°C. Prior to lysis by French press, cells were resuspended in TNI buffer, and protease activity was inhibited by addition of 1 mM phenylmethylsulfonyl fluoride (PMSF) and 1 mM diisopropylfluorophosphate (DFP). The extract was cleared by centrifugation for 1 h at 25000 × g. Peak fractions of Ni-nitrilotriacetic acid (Ni-NTA) agarose affinity chromatography eluted with 20 mM Tris, 300 mM NaCl, 100, 200, or 400 mM imidazol, and 5% (vol/vol) glycerol were pooled, and the pooled fractions were desalted using Sephadex G25 gel filtration (Amersham Bioscience, Uppsala) and buffered in 100 mM triethanolamine hydrochloride (TEA-Cl), pH 7.4.

The molecular weight of PGK^His^ was determined using gel filtration and by cross-linking experiments. For gel filtration, a Bio-Prep SE-1000/17 column (BioRad, Richmond, VA, USA) was used and calibrated with Gel Filtration Standard (BioRad) containing thyroglobulin (670 kDa), bovine γ - globulin (158 kDa), chicken ovalbumin (44 kDa), equine myoglobin (17 kDa), and vitamin B_12_ (1.35 kDa). The buffer used contained the optimal conditions for activity of PGK plus 100 mM TEA-Cl pH 7.4). To determine the PGK elution volume, two gel filtrations were performed, one in a mix with the indicated protein standards containing 2 mg/ml of PGK and a second with 4 mg/ml of PGK. Protein elution time was measured at 280 nm. Eluting PGK^His^ was collected in 0.5 ml fractions and confirmed by enzyme activity measurement.

### Assay conditions

The phosphoglycerate kinase activity was determined with purified enzyme and cell-free extracts of WT(pEKEx3) and WT(pEKEx3-*pgk*). Cell-free extracts were prepared according to Stansen *et al.*[57]. In vitro enzyme assays were carried out spectrophotometrically in a coupled assay in which, product of the first reaction catalyzed by *Cg*PGK, namely 1, 3 - bisphosphoglyerate is reduced by the second enzyme glyceraldehyde-3-phosphate dehydrogenase, which uses NADH. Assays were conducted in 100 mM triethanolamine-HCl buffer (pH-7.4) containing 1 mM EDTA, 2 mM MgSO_4_, 1 mM ATP, 10 mM 3-PGA, 0.2 mM NADH, 10 U/ml of GAPDH (rabbit muscle). Approximately 30 ng of PGK^His^ was used for each assay. The enzyme activity was measured by following the decrease in absorbance at 340 nm due to oxidation of NADH [14]. All spectrophotometric measurements were carried out using a Shimadzu UV-1202 spectrophotometer (Duisburg, Germany) at 30°C, NAD^+^ formation was followed at λ = 340 nm (ϵ_340nm_ = 6.3 mM^-1^ cm^-1^). Kinetic parameters were calculated using Michaelis–Menten kinetics. One unit (U) of enzyme activity is defined as 1 μmol × min^-1^ × mg^-1^ of protein. PEPCx was assayed at 30°C in 1 ml of 100 mM Tris–HCl buffer (pH 8.0) containing 10 mM MgSO4, 25 mM NaHCO3, 1 mM dithiothreitol (DTT), 0.2 mM NADH, 10 U of malate dehydrogenase, and 8 mM PEP. One unit (U) of activity is defined as 1 mmol of NADH consumed per min. TPI was assayed at 30°C in 1 ml of 300 mM triethanolamine buffer (pH 7.6), 0.2 mM NADH, 2 U of glycerolphosphate dehydrogenase, 5 mM glyceraldehyde-3-phosphate as the substrate. The decrease of NADH was monitored at 340 nm.

To determine the pH-optimum TEA-Cl was replaced by the following buffers (50 mM): acetate (pH 5.0-6.0), phosphate (pH 6.0-7.0), TEA-Cl (pH 7.0-9.0), and glycine-NaOH (pH 9.0-10.0) under standard conditions. The pH was adjusted at room temperature. The effect of metal ions and EDTA on kinase activity was measured under standard conditions in the presence of Zn^2+^, Ca^2+^, Co^2+^, Cd^2+^, Cu^2+^, Mg^2^, Fe^2+^, Mn^2+^, Ni^2+^, at 0.5 and 1 mM final concentration in the reaction mixture. For determination of optimum temperature, the reaction mix was allowed to equilibrate for 5 min at each temperature point.

### Overexpression of *pgk* in production strains

Cells were removed from the culture samples by centrifugation for 10 min at 14,000 × g and supernatant was analyzed using a high-pressure liquid chromatography system (HPLC, 1200 series, Agilent Technologies). L-lysine, L-ornithine and L-arginine concentrations were determined by automatic precolumn derivatization with ortho-phthaldialdehyde and reversed-phase high-performance liquid chromatography (RP-HPLC) with fluorimetric detection (excitation at 230 nm; emission at 450 nm). The buffer gradient consisted of 0.1 M sodium acetate, pH 7.2 (with 0.03% sodium azide), as the polar phase and methanol as the nonpolar phase. L-asparagine was used as internal standard for L-lysine, L-ornithine and L-arginine respectively.

### Computational analysis

Sequence comparisons were carried out with protein sequences obtained from the NCBI database (http://www.ncbi.nlm.nih.gov) using CLUSTAL W [60], the alignment was formatted using BoxShade and phylogenetic trees were constructed using the neighbor-joining method [61] with 1,000 bootstrap replicates, the tree was rooted against phosphoglycerate kinase of *E. coli* (data not shown). Besides the protein sequence of *C. glutamicum* PGK (Cg1790), protein sequences of characterized PGKs from the following organisms were used: *E. coli* (b2926)*, Methanothermus ferviduds* (Mfer_0156), *Pyrococcus woesi* (*Accession ID* P61884.1), *Sulfolobus solfataricus* (CAA 56459), *Trypanosoma brucei (*AAA32120.1).

## Abbreviations

PGK: 3-phosphoglycerate kinase; PGKHis: PGK with histidine tag; 3-PG: 3-phosphoglycerate; EV: Empty vector; GAP: Glyceraldehyde 3-phosphate; GAPDH/GapA: Glyceraldehyde-3-phosphate dehydrogenase; HPLC: High performance liquid chromatography; IPTG: Isopropyl β-D-1-thiogalactopyranoside; ATP: Adenosine tri phosphate; GTP: Guanosine tri phosphate; AMP: Adenosine mono phosphate; ADP: Adenosine di phosphate; SDS-PAGE: Sodium dodecyl sulfate polyacrylamide gel electrophoresis; TEA-Cl: Triethanolamine hydrochloride.

## Competing interests

The authors do not declare competing interests.

## Authors’ contributions

VFW and GKR designed the experiments. GKR conducted the experiments, analyzed the results, and wrote the manuscript. VFW reviewed and revised the manuscript. Both authors read and approved the final manuscript.
